# Automated stomata detection in oil palm with convolutional neural network

**DOI:** 10.1038/s41598-021-94705-4

**Published:** 2021-07-26

**Authors:** Qi Bin Kwong, Yick Ching Wong, Phei Ling Lee, Muhammad Syafiq Sahaini, Yee Thung Kon, Harikrishna Kulaveerasingam, David Ross Appleton

**Affiliations:** 1 Sime Darby Plantation Technology Centre Sdn Bhd, Serdang, Selangor Darul Ehsan Malaysia; 2 Department of Biology and Biotechnology, Faculty of Science and Technology, National University of Malaysia, Bangi, Selangor Darul Ehsan Malaysia

**Keywords:** Machine learning, Stomata

## Abstract

Stomatal density is an important trait for breeding selection of drought tolerant oil palms; however, its measurement is extremely tedious. To accelerate this process, we developed an automated system. Leaf samples from 128 palms ranging from nursery (1 years old), juvenile (2–3 years old) and mature (> 10 years old) were collected to build an oil palm specific stomata detection model. Micrographs were split into tiles, then used to train a stomata object detection convolutional neural network model through transfer learning. The detection model was then tested on leaf samples acquired from three independent oil palm populations of young seedlings (A), juveniles (B) and productive adults (C). The detection accuracy, measured in precision and recall, was 98.00% and 99.50% for set A, 99.70% and 97.65% for set B, and 99.55% and 99.62% for set C, respectively. The detection model was cross-applied to another set of adult palms using stomata images taken with a different microscope and under different conditions (D), resulting in precision and recall accuracy of 99.72% and 96.88%, respectively. This indicates that the model built generalized well, in addition has high transferability. With the completion of this detection model, stomatal density measurement can be accelerated. This in turn will accelerate the breeding selection for drought tolerance.

## Introduction

The key to sustainable agriculture and managing the impact of climate change is selective breeding. Through selective breeding, the global increase in demand for food can be achieved through improvement in yield per unit area, thereby not requiring new agricultural land. Particularly in the case of oil palm, this means a halt to deforestation, therefore conserving the habitats for biodiversity. In the past, selective breeding has been performed using phenotypic selection or limited marker assisted selection. The advancement in genomic sciences has allowed for high throughput, whole genomic marker discovery and deployment for selective breeding. Whole genomic marker-based selection is known as genomic prediction or selection^1^, which has seen a lot of success from cattle to maize breeding^2,3^. Genomic selection is usually conducted by regressing the trait values against the genotypes, resulting in a model that can be used to predict for individuals with known genetic information but unknown trait performance. Since the conceptual introduction of it in oil palm^4^, genomic selection has since been assessed and applied in different oil palm breeding programs^5,6^.

Even though high throughput genotyping has been made possible^7,8^, the phenotyping process usually still relies on manual measurement and recording. This, together with the fact that breeders are usually interested in multiple traits, creates a phenomenon known as phenotyping bottleneck^9^. Time taken for phenotyping essentially limits progress in plant breeding programs, including drought tolerance^10^. Therefore, it is crucial to automate phenotyping processes in order to accelerate selective breeding. The first step towards automating the entire phenotyping process often lies in image analytics and machine learning. Essentially, once an image of a plant has been captured through a device, machine learning can be employed to automate the measurement, counting or classification of the objects in the image. Applications developed based on this include Deep Plant Phenomics, which is capable of leaf counting and mutant classification^11^.

One of the key traits of interest for oil palm is drought tolerance, particularly related to minimizing climate change impact. There are many methods to define drought tolerance, including taking a range of leaf measurements^12^. The most direct measurement of drought tolerance is based on water-use efficiency and yield under water-limiting conditions^13^. Another alternative is to measure the stomatal density. Biologically, stomata are an important “gate” for both gas exchange (CO2) of the leaf during photosynthesis and water vapor during transpiration^14^. Previous studies have shown that photosynthesis and efficiency of water usage in plants were affected by the responsiveness, speed and size of stomata^15,16^. Low stomatal density has been reported to increase drought tolerance in many plant species, including barley^17^, rice^18^, wheat^19^ and arabidopsis^20^. In addition, regulation capacity of stomatal traits including size and density were also identified as key traits to select for drought tolerance^21^. With that, the focus of this study was to develop an automated method of detecting and counting stomatal density using convolutional neural network (CNN).

## Methodology

### Sample collection

A total of 128 leaf samples were obtained from advanced commercial oil palms, collected from Sime Darby Plantation nurseries and estates in Carey Island and Banting, Selangor, Malaysia. Among these 128 samples, 42 were collected from nursery seedlings (1 years old (yo)), 43 were from juvenile palms (2–3 yo) planted in estates, and 43 were collected from adult palms (> 10 yo) in commercial estates. For seedlings, bifurcated leaf number 3 was collected, whereas middle leaflets from frond number 17 were collected for juvenile and adult palms.

### Image capture and stomatal characterization

The bottom surfaces of leaf samples were cleaned with distilled water and air-dried. A thin layer of nail vanish was applied at the middle part of the oil palm leaf (approximately 1 cm × 3 cm in size). After air-drying, a layer of transparent tape was applied firmly on top of the dried nail vanish. The layer of cellofoam tape was then peeled with the nail vanish stuck together and transferred to a clean slide, generating the stomata imprint^22^. Micrographs of size 1800 µm × 1400 µm were acquired with a 10× objective lens using the RGB-mode illumination on an EVOS FL Auto (Thermo Scientific, US) microscope.

Stomatal density and size were recorded and measured manually from the micrographs. Density was counted as the number of stomata objects in 1 mm^2^. The stomatal size in this study was defined as the horizontal length measured from one stoma end to the other. The average stomatal size was estimated based on the size of 150 randomly selected stomata across all images for that particular age group. Statistical *t*-test was carried out to determine significant differences for stomatal density and size measured between each age group.

### Preprocessing

The acquired 128 micrographs were of resolution 2048 × 1536. Each image was cropped into 12 independent 516 × 512 tiles, resulting in 1536 tiles. Of these tiles, 51 from young seedlings, 171 from juvenile palms, and 88 from adult palms were manually selected to build the CNN stomata detection model. The tiles were manually annotated for stomata objects using labelImg software^23^. Overall, 20,809 stomata objects were annotated.

The acquired tiles were manually inspected, and 193 tiles of the training set were subjected to augmentation using an in-house Python script. Blurring of the images was done using both “bilateralFilter” and “GaussianBlur” functions from OpenCV library^24^. Brightness of the selected tiles were also altered (brightened and darkened) by a factor of 2. After augmentation, there were 49,837 stomata objects.

### Model building

**Figure 1:**
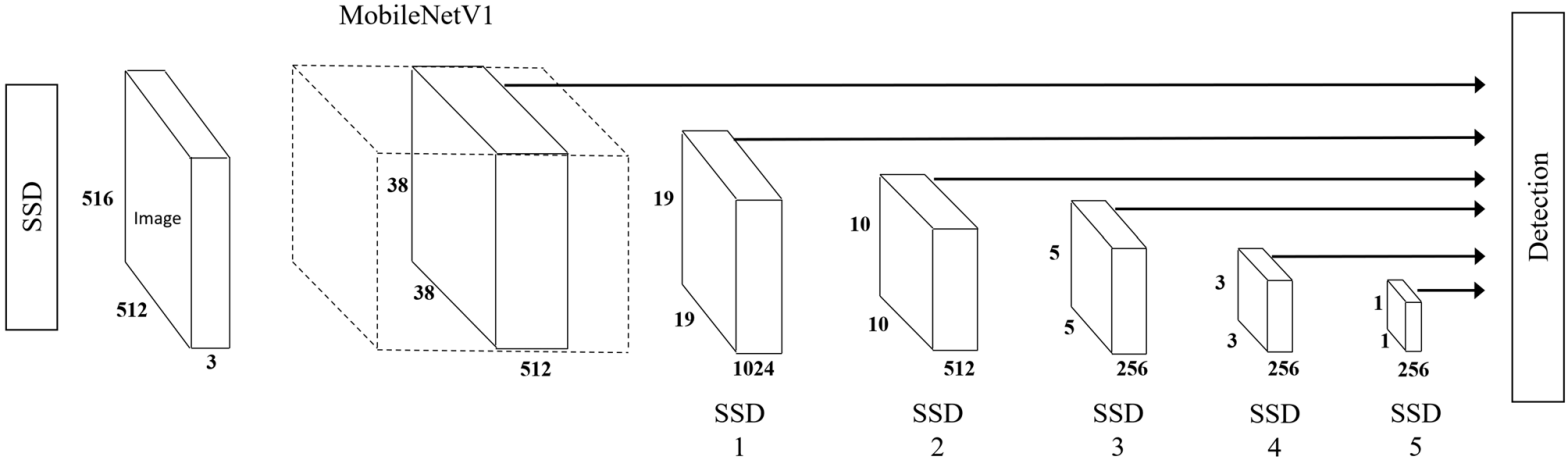
Diagram showing SSD^28^ using MobileNetV1^27^ as backbone.

This study was carried out using Google Cloud Platform’s Compute Engine, with the specification of Tesla T4 GPU, 128 GB RAM and 24 CPU. The stomata detection model was trained using tensorflow^25^ library’s object detection API^26^. MobileNet Version 1 (MobileNetV1) is a light weight deep neural network suitable for mobile vision applications, based on a streamlined architecture with depth-wise (dw) separable convolutions^27^. Table 1 summarized the MobileNetV1 architecture. Single Shot Multibox Detector (SSD) is a deep network based accurate object detector that does not resample pixels or features while forming bounding boxes^28^. Instead of the original VGG-16 architecture^29^, the MobileNetV1-based Single Shot Multibox Detector (SSD) (Fig. 1) model trained on COCO dataset^30^ was used in this study. The model was retrained to detect stomata through transfer learning, with the initial weights being MobileNetV1’s pretrained weights.

**Table 1:** MobileNet body architecture^27^.

Type/stride	Filter shape	Input size
Conv/s2	3 × 3 × 3 × 32	224 × 224 × 3
Conv dw/s1	3 × 3 × 32 dw	112 × 112 × 32
Conv/s1	1 × 1 × 32 × 64	112 × 112 × 32
Conv dw/s2	3 × 3 × 64 dw	112 × 112 × 64
Conv/s1	1 × 1 × 64 × 128	56 × 56 × 64
Conv dw/s1	3 × 3 × 128 dw	56 × 56 × 128
Conv/s1	1 × 1 × 128 × 128	56 × 56 × 128
Conv dw/s2	3 × 3 × 128 dw	56 × 56 × 128
Conv/s1	1 × 1 × 128 × 256	28 × 28 × 128
Conv dw/s1	3 × 3 × 256 dw	28 × 28 × 256
Conv/s1	1 × 1 × 256 × 256	28 × 28 × 256
Conv dw/s2	3 × 3 × 256 dw	28 × 28 × 256
Conv/s1	1 × 1 × 256 × 512	14 × 14 × 256
$5\times \genfrac{}{}{0pt}{}{Conv dw/s1 }{Conv/s1}$	3 × 3 × 512 dw	14 × 14 × 512
	1 × 1 × 512 × 512	14 × 14 × 512
Conv dw/s2	3 × 3 × 512 dw	14 × 14 × 512
Conv/s1	1 × 1 × 512 × 1024	7 × 7 × 512
Conv dw/s2	3 × 3 × 1024 dw	7 × 7 × 1024
Conv/s1	1 × 1 × 1024 × 1024	7 × 7 × 1024
Avg Pool/s1	Pool 7 × 7	7 × 7 × 1024
FC /s1	1024 × 1000	1 × 1 × 1024
Softmax /s1	Classifier	1 × 1 × 1000

80% of the tiles were used for the model training set, while 20% were used as a validation set. From the 49,837 stomata objects, 39,877 were used for model training, while 9960 were used for validation. During training, L2 regularization was carried out to prevent overfitting. RMSprop and momentum optimizers were also used during training to reduce training time required. After a few rounds of manual running and adjustments, the initial learning rate was set at 0.003, and the decay factor was set at 0.95, decay steps at 100. Number of classes was set at 1 and batch size was set 20. The detection score threshold was set at 0.2.

The training progress was monitored through the use of Tensorboard^25^. The overall loss function ($L$) used was the combination of classification loss and the weighted sum of the localization loss, which is represented as follow:

$$L=\frac{1}{N}\left({L}_{c}+\alpha {L}_{l}\right),$$

where ${L}_{c}$, the classification loss is essentially the softmax loss over the different classes^28^, ${L}_{l}$, the localization loss, is the smooth L1 loss^31^ between the predicted bounding box and the ground truth box, *N* is the number of matched default boxes^28^ and $\alpha$ is the weight factor. The validation metric used to measure the performance of the model was mean average precision (mAP). The model was only saved/updated if it was performing better than the previous checkpoints. Early stopping was employed to prevent model overfitting.

### Application

Three independent datasets, consisting of 55 leaf samples from 1 yo seedlings (set A), 135 leaf samples from 2 to 3 yo palms (set B) and 100 samples from > 10 yo palms (set C) were collected and used as the application/test set. Similarly, the micrographs were split into tiles. In this case, however, they were split into 20 overlapping (120 pixels overlapping horizontally and 40 pixels vertically) tiles, resulting in 5800 tiles. The stomata objects on these tiles were detected using the model built previously with detection score threshold set at 0.2. After prediction, the bounding boxes generated were overlaid on the full-size micrographs using a Python script. Non-maximum suppression was used to solve the issue of overlapping bounding boxes caused by splitting, with the threshold set as 0.01. The acquired results were manually inspected and validated. Accuracy of the prediction was measured in both precision and recall. Precision was defined as true positives divided by total detected cases in the image, whereas recall was defined as true positives divided by total stomata in the image. As this dataset was not annotated, both precision and recall were calculated manually. In this case, correct detections were defined as bounding boxes overlapping with the stomata objects.

### Challenge/limitation

Another 20 independent leaves were sampled from palms sharing the same genetic background as Set C. However, in this case the micrographs were captured under the 4× objective lens of an ECLIPSE Ci-L Nikon, Japan microscope. Stomata images of size 1589 µm × 1192 µm were captured from this step. The model was cross applied onto the current dataset, hereby referred to as Set D.

### Statement of consent

This study on oil palm complies with relevant institutional, national, international guidelines and legislation. All samples collected are maintained and belong to Sime Darby Plantation R&D, Malaysia.

## Results

### Stomatal characterization across different palm developmental stages

**Figure 2:**
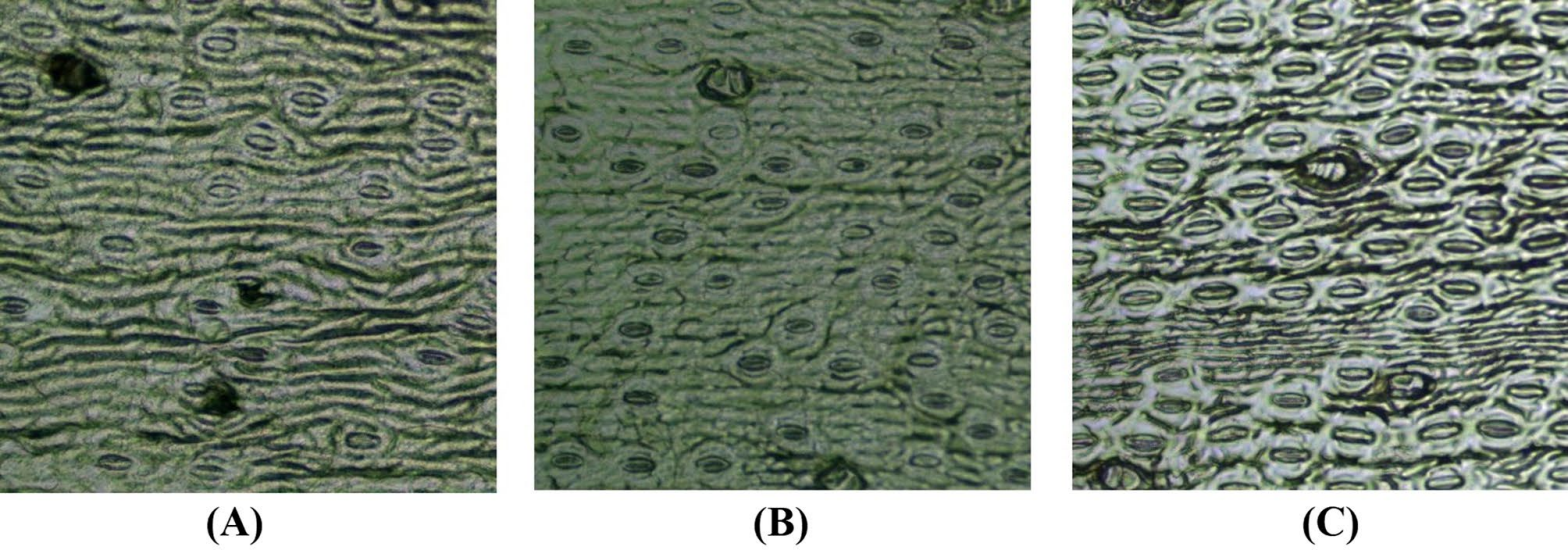
Representative stomata tiles for three palms’ developmental stages collected in this study. (**A**) 1 yo palm seedling, (**B**) 2–3 yo juvenile palm, (**C**) > 10 yo adult palm.

The average stomatal count in an image was 107 ± 12 (mean ± standard deviation) for the 1 yo palms (Set A) and 200 ± 18 for 2–3 yo palms (Set B) and 208 ± 16 for > 10 yo palms (Set C). Besides having the highest stomatal count, the stomatal size of Set C was also the largest, at 43.01 ± 3.51 µm, as compared to Set A and Set B (31.82 ± 2.64 µm and 34.41 ± 2.67 µm, respectively) (Table 2). Example stomata tiles for all three stages can be found in Fig. 2.

**Table 2:** Stomatal count and size across different palm age groups.

Set	Palm age (yo)	Count mean (/mm^2^)	Count sd (/mm^2^)	Size mean (µm)	Size sd (µm)
A	1	107^α^	12	31.82^α^	2.64
B	2–3	200^α^	18	34.41^α, β^	2.67
C	> 10	208	16	43.01^β^	3.51

The stomatal counts and standard deviations (sd) were rounded to its’ nearest integer. Significant differences at p-value < 0.05 were represented as α for A versus B, and β for B versus C.

### Model and application

**Figure 3:**
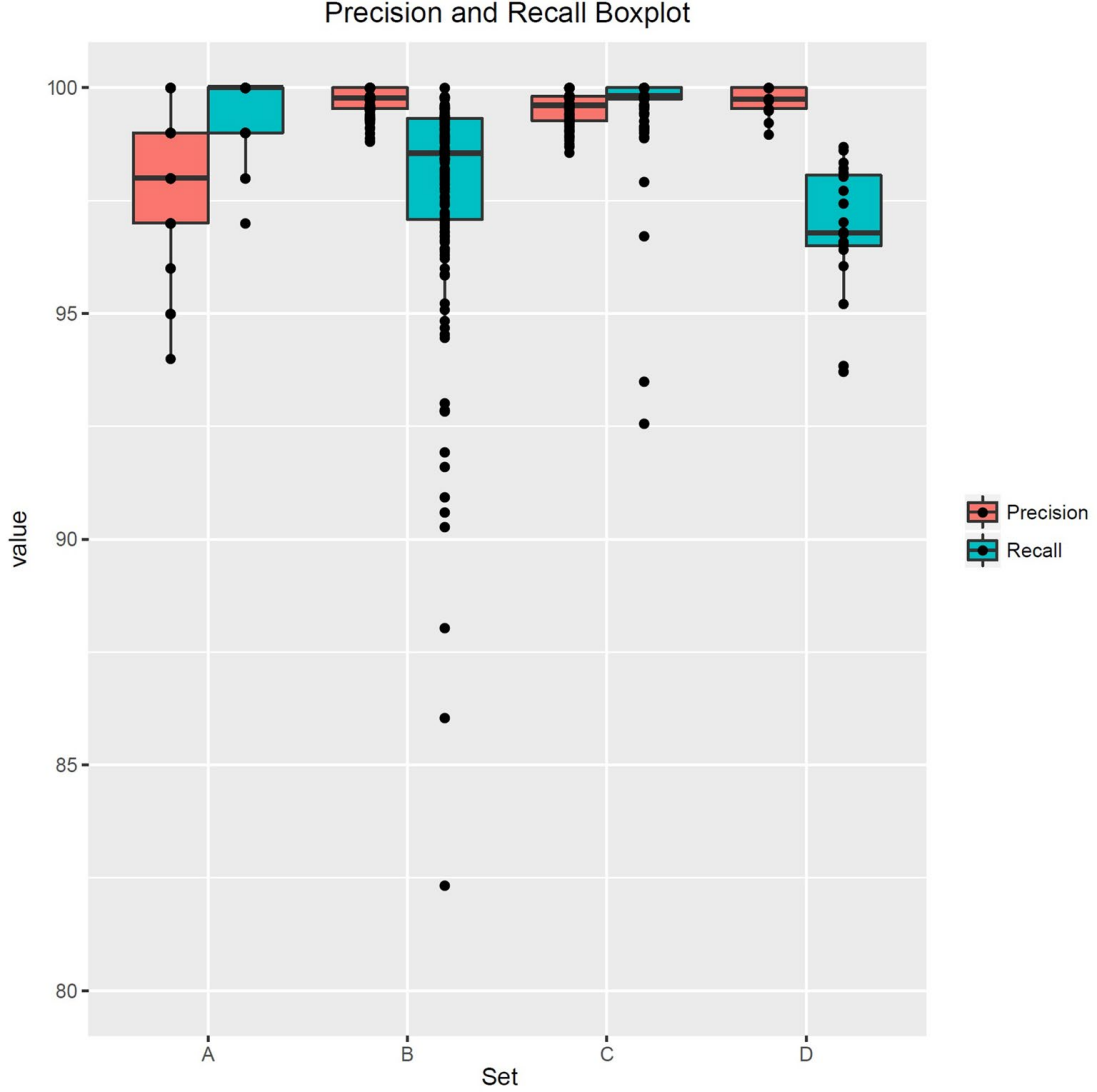
Precision and recall boxplot for four test sets (A, B, C and D).

**Figure 4:**
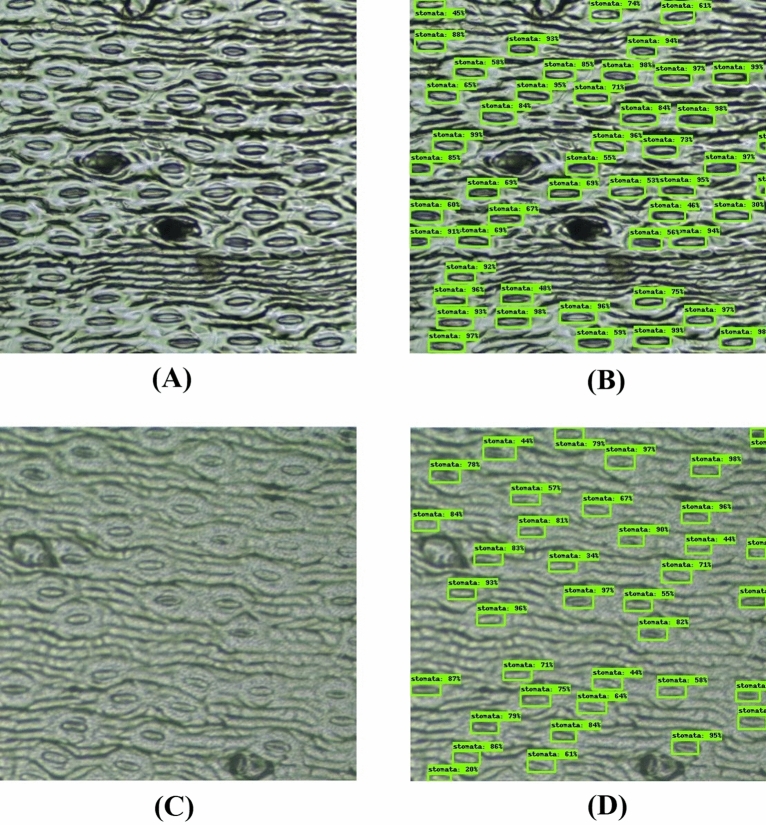
Representative tiles before and after automated detection using developed stomata model. The detected stomata were labelled in green bounding boxes together with a detection score on top. (**A**) Clear tile. (**B**) Stomata detected on clear tile. (**C**) Unfocused tile. (**D**) Stomata detected on unfocused tile.

**Figure 5:**
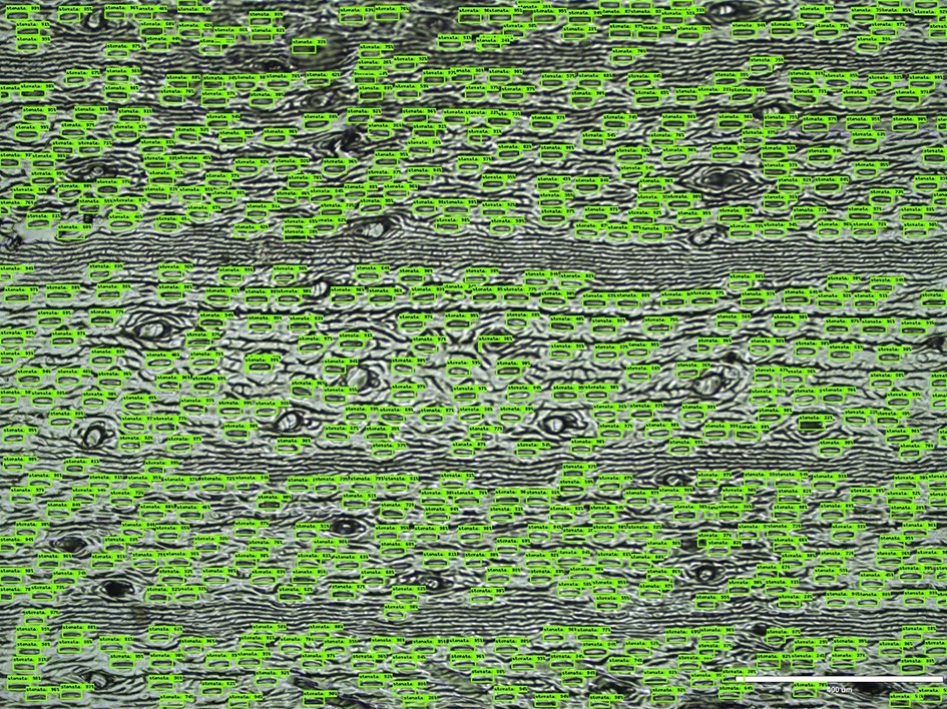
Stomata detection on entire microscopic image. The detected stomata were labelled in green bounding boxes together with a detection score on top.

The mean average precision (mAP) of the detection model was 96% (with intersection over union (IoU) threshold set at 0.5) when training was stopped. At this point, the overall loss value was 1.34. The model built was then applied on independent test sets (A, B, C and D). Figure 3 shows the boxplot summarizing precision and recall values acquired for all four test sets. For the image with the highest stomatal density (Supplementary Fig. S1), both precision and recall calculated were 99.83%. As for the image with the lowest stomatal density (Supplementary Fig. S2), precision was 98.91% and recall was 99.45%. The average precision acquired when testing the model on set A was 98.00 ± 1.40% (mean ± standard deviation), with the corresponding recall being 99.50 ± 0.70%. Comparatively, the average precision acquired for set B was 99.70 ± 0.29% and the recall was 97.65 ± 2.76%. The image with the lowest recall value (82%) can be found in Supplementary Fig. S3. As for set C, the model achieved average precision of 99.55 ± 0.37%, and recall of 99.62 ± 1.05%. The cross application of the model onto set D dataset exhibited precision of 99.72 ± 0.29% and a slight drop in recall of 96.88 ± 1.40%. The worst performing image is included as Supplementary Fig. S4. In addition, examples of raw and annotated tiles are shown in Fig. 4, including successful application on an unfocused image (Fig. 4D). Figure 5 shows an example of a fully annotated stomata image using the built model.

## Discussion

To the best of our knowledge, this is the first CNN-based oil palm stomata detection model reported. In other plant species such as grapevine^32^ and oak^33^, stomata detection and morphological feature estimation have been automated through cascade object detector utilizing feature extractors such as histogram of oriented gradients. In oak, the estimated precision and recall were 95% and 85%, respectively. As for grapevine, the precision and recall were 92% and 79%, respectively. In these methods, parameters associated with these feature extractors were manually defined. Though the precision values acquired from these studies were very high, the recall values were lower by comparison. Compared to these methods that use specific feature engineering methods, a more recent methodology of automating stomata detection using multiple feature extraction techniques and learning methods was introduced in maize^34^, achieving 97.1% detection accuracy (measured in precision). This study also highlighted the use of deep learning features for stomata detection application. StomataCounter^35^ was one of the earliest efforts in building CNN-based stomata detection model. The model built reached precision of 99% and recall of 93%, indicating that CNN performed better than other methods. It was also noted in the study that model accuracy would decrease significantly when cross-applied on images from another species.

Our current study focused on developing an automated stomata detection model specifically for oil palm. For this purpose, we have selected oil palm samples from different developmental stages or age groups, ranging from young seedling to adult. One of the concerns with only using training dataset images from a single age group was that the resulting detection model might not generalize well across other age groups. From the stomatal characterization performed, we observed significant difference in stomatal density between seedling and juvenile palms. Although the stomatal density of adult palms was the highest, it was not statistically different from juvenile palms. On the other hand, stomatal size measurements showed significant differences across all age groups. It is noted, however, that the size difference observed between juvenile and seedling was far lesser when compared to the difference between adult and juvenile. This indicates that the juvenile palms’ (age 2–3 yo) stomatal profile captures the transitional stages of stomata from seedling to adult. By combining these images with images acquired from both seedling and adult palms, the resulting model would be capable of detecting stomata across all age groups in oil palm. Thus, not only can this model be used for yield-related studies in adult palms, it can also be used early screening of drought tolerant palms in nursery.

In general, the accuracy acquired in this study was similar to a previous publication on stomata detection using CNN^35^, with the acquired recall values reported here being slightly higher. This is probably attributed to the preset condition that our model was both trained and expected to only be used for oil palm stomata micrographs, as compared to a general model^35^ that is expected to work across different species. The high recall and precision values indicated that the model seldom miss stomata, nor misclassify non-stomata objects. In fact, compared to other non-CNN methods^32,33^, CNN-based detection models achieved far better recall values, indicating that they generalized better. This is probably because parameters involved in CNN were determined through training and not manually decided. In addition, CNN has a deeper architecture which provides exponentially more expressive capability, and its’ hierarchical feature representation enables multilevel representation from pixel to high-level features^36^. Although CNN models generalize better, the required condition to build CNN model is that the number of samples needs to be large, which was a limitation faced in previous grapevine and oak publications^32,33^. As our final goal is to develop a mobile application that enables field stomatal count phenotyping, the MobileNet CNN architecture, which is known to be light weight was selected. Other CNN architectures, such as VGGNet^29^ were not assessed, which can be seen as a limitation of this study.

With large volume of data comes the problem of data quality. Two problems faced when working on micrographs in this study were inconsistent brightness and unfocused images, which were predominantly faced during set B application (example shown in Fig. 4C). This is potentially caused by uneven stomata imprint or non-optimal microscope brightness and contrast settings. In moving towards high-throughput phenotyping using micrographs, this represents one of the practical issues that needs to be solved. Another option is to develop a robust detection model. Instead of discarding these low-quality images, the CNN model was also trained on them as well. In fact, to increase the number of low-quality images, some of the high-quality images were artificially altered to be unfocused and darkened/lightened in a process known as augmentation. The augmentation process also increases the number of annotated images for training, which is often regarded as a taxing and expensive process in developing an object detection model^37,38^. With this, the resulting model was also capable of detecting stomata on most low-quality images (example shown in Fig. 4D). Another method to reduce manual annotation used in this study was only annotating selected tiles from a full-size image instead of annotating every single one. With tiles within the same image sharing similar background properties, it was rather redundant to annotate all of them. Instead, representing every training image with a few tiles allows for building of a more robust model with lesser effort.

Although the resulting oil palm stomata detection model performed well, it was not without limitation. In rare cases, we observed low recall but high precision, which indicates failure of the model in detecting certain stomata objects. Upon inspection of these images (Supplementary Figs. S3, S4), it was found that the undetected stomata fall within the image regions that had a combination of unfocused stomata objects, low-contrast or noisy background. Further improvements to the model can be made through training on images with similar properties. In addition, the stomata objects that were found near the edges of the images were sometimes undetected. A potential solution that can be implemented is to subset the test image by ignoring the last few pixels both vertically and horizontally^33,35^.

One of the key criteria when building a predictive model is that the training dataset must be representative of the test/application set by having similar distribution^37^. In practice, however, it is impractical to assume that images acquired under different microscope and different conditions will share the same underlying distribution. As such, the detection model was tested on another set of images (D) acquired from a different microscope under different conditions such as lighting and image size. Set D achieved the similar precision with B and C at 99.72%, indicating that the model was able to detect stomata in the images from different microscope with low false positives. Despite the slight drop in recall to 96.88%, overall the model performed rather well in this challenge test. In other words, the model can be cross-applied to micrographs generated using other microscopes. Yet, the slight drop in recall also indicates that the detection model needs to be continuously be improved using images acquired under different conditions.

With our main goal being to develop drought tolerant yet productive palms, commercial populations developed from key breeding programs were selected for this study. Stomata detection from micrographs was identified as one of the key time-consuming steps. To ease this phenotyping process, this stomata detection CNN model was developed. The resulting model showed promising result, particularly on adult palms. From our experience, manual stomata counting of a single full image can take up to 5 min, and automating stomata counting on a large dataset can save months of manpower. Without the limitation in phenotype analysis, the result acquired using this model can be used for genome-wide association study or quantitative trait locus analysis, which identifies key genes controlling drought tolerance and requires a large dataset of palms to be used. Also, high-throughput phenotyping methods will accelerate genomic selection^5,6^, thereby reducing time required for breeding and selection. As an initial step towards automating stomata phenotyping in oil palm, future models will need to not only detect stomata, but also segment out and measure their sizes and openings. The model can then be incorporated into a portable device for field phenotyping. Given the high accuracy observed, other CNN models can be developed in the future for many other commercially useful traits and for precision agriculture, for example automatic palm detection from drone/satellite images and disease classification in estates or nurseries.

## Conclusion

This paper describes the development of the first oil palm CNN-based stomata detection model using leaves collected from palms of different ages. The resulting model showed very high accuracy (99.08% precision and 98.92% recall) when tested on three different age groups. The model also demonstrated high transferability, achieving high accuracy (99.72% precision and 96.88% recall) when cross-applied onto micrographs acquired under different conditions.

## Supplementary Information

Supplementary Information.
