# Supplementary material for: Automated stomata detection in oil palm with convolutional neural network
Source: Sci Rep. 2021 Jul 26;11:15210. doi: 10.1038/s41598-021-94705-4 (PMC8313554; doi:10.1038/s41598-021-94705-4)
Supplement: Supplementary file 1 — Supplementary Information. [file 41598_2021_94705_MOESM1_ESM.docx]

Automated Stomata Detection in Oil Palm with Convolutional Neural Network

Qi Bin Kwong^1*^, Yick Ching Wong^1^, Phei Ling Lee^2^, Muhammad Syafiq Sahaini^2^, Yee Thung Kon^1^, Harikrishna Kulaveerasingam^1^, David Ross Appleton^1^

^1^ Sime Darby Plantation Technology Centre Sdn Bhd, Serdang, Selangor Darul Ehsan, Malaysia

^2^ Department of Biology and Biotechnology, Faculty of Science and Technology, Universiti Kebangsaan Malaysia

* Correspondence: Qi Bin Kwong (kwong.qi.bin@simedarbyplantation.com)

Supplementary Information


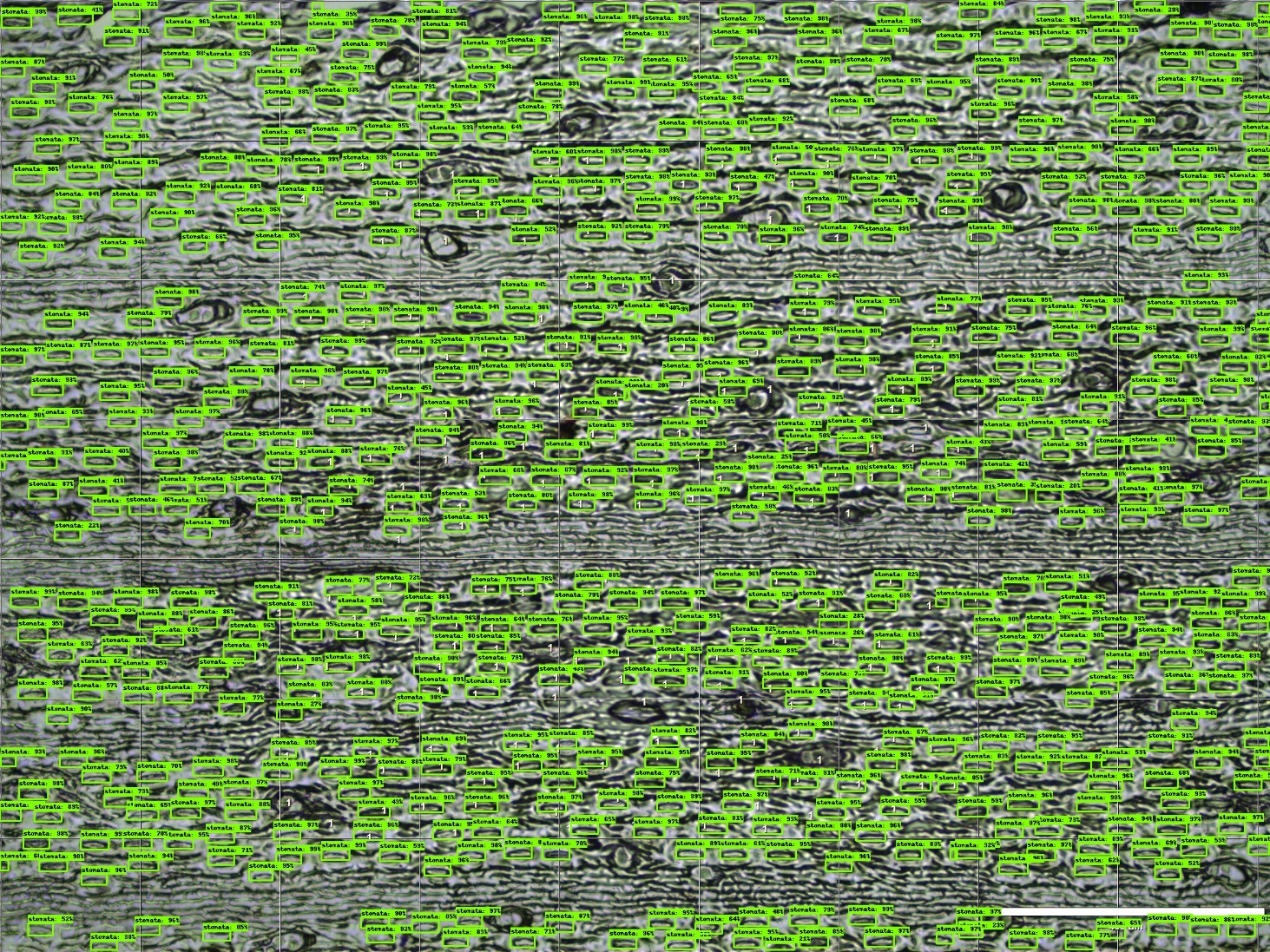


Supplementary Figure 1. Application of stomata detection model on image with the highest stomata number. Both precision and recall calculated were 99.83%.


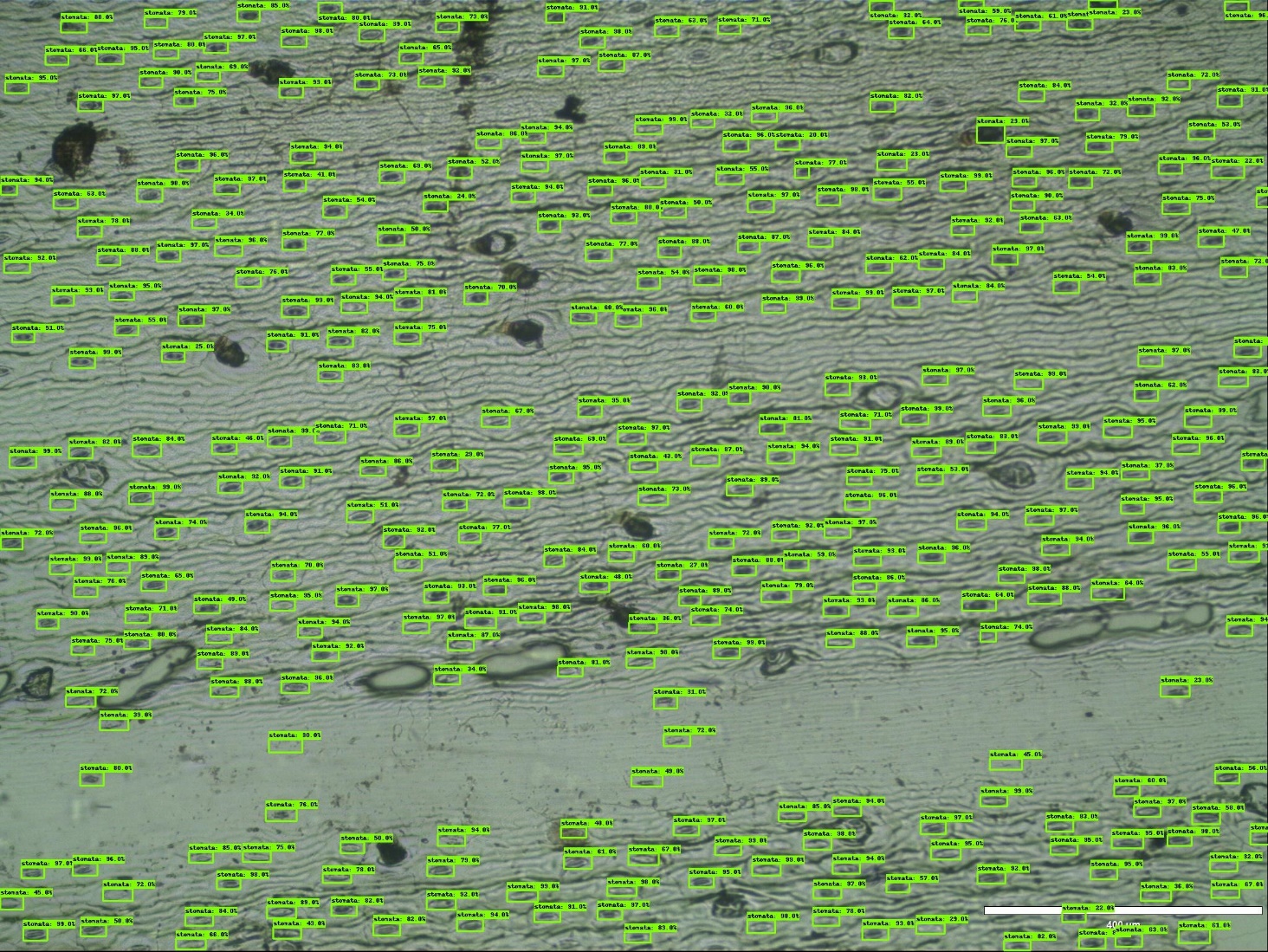


Supplementary Figure 2. Application of stomata detection model on image with the lowest stomata number. Precision was 98.91% and recall was 99.45%.


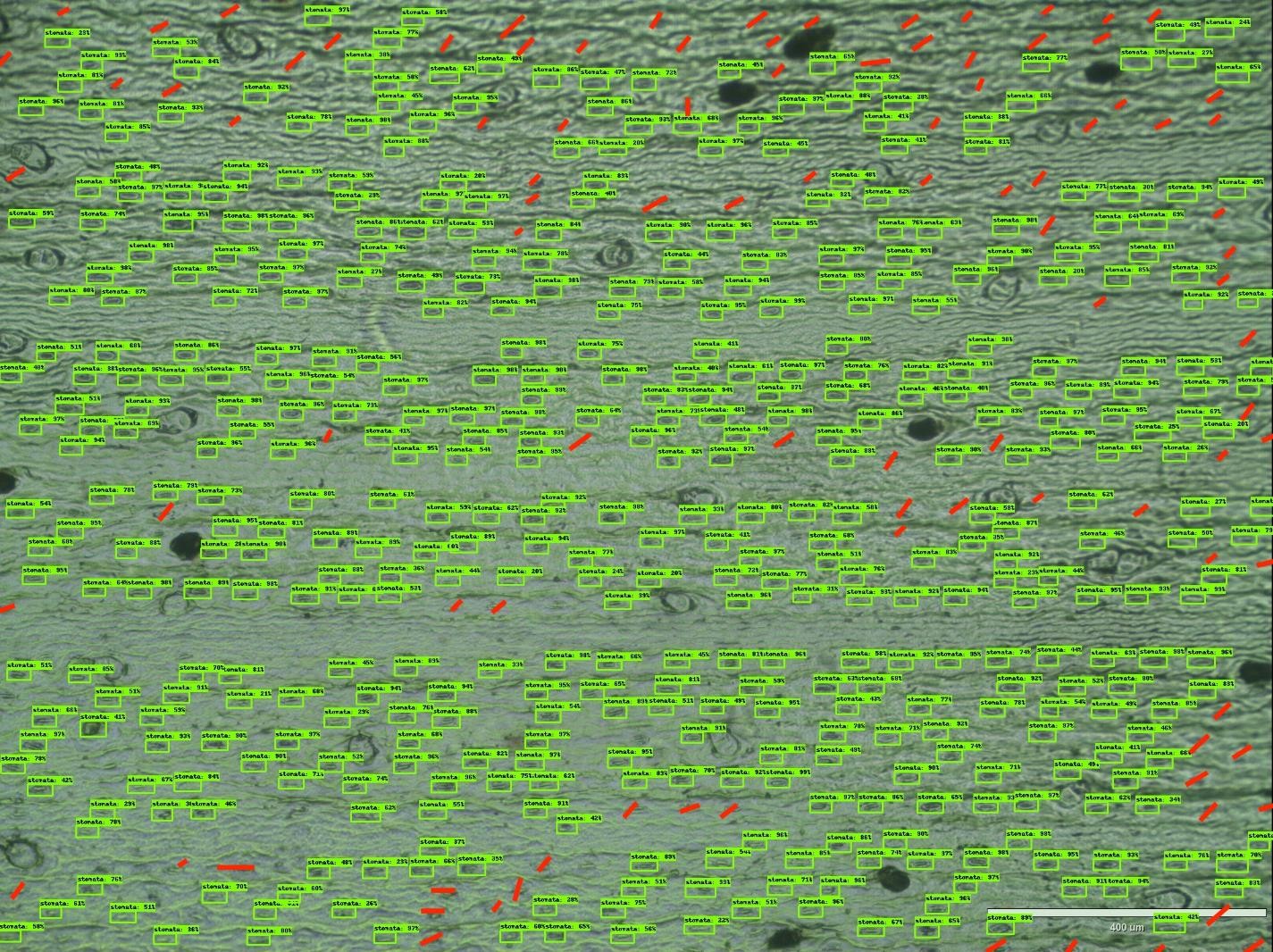


Supplementary Figure 3. Extreme case with lowest accuracy, caused by unfocused and low contrast quality stomata objects for Set B. The stomata detection precision for this micrograph was 100%, and the recall was 82%, indicating that some stomata were not detected. The undetected stomata were manually marked in red.


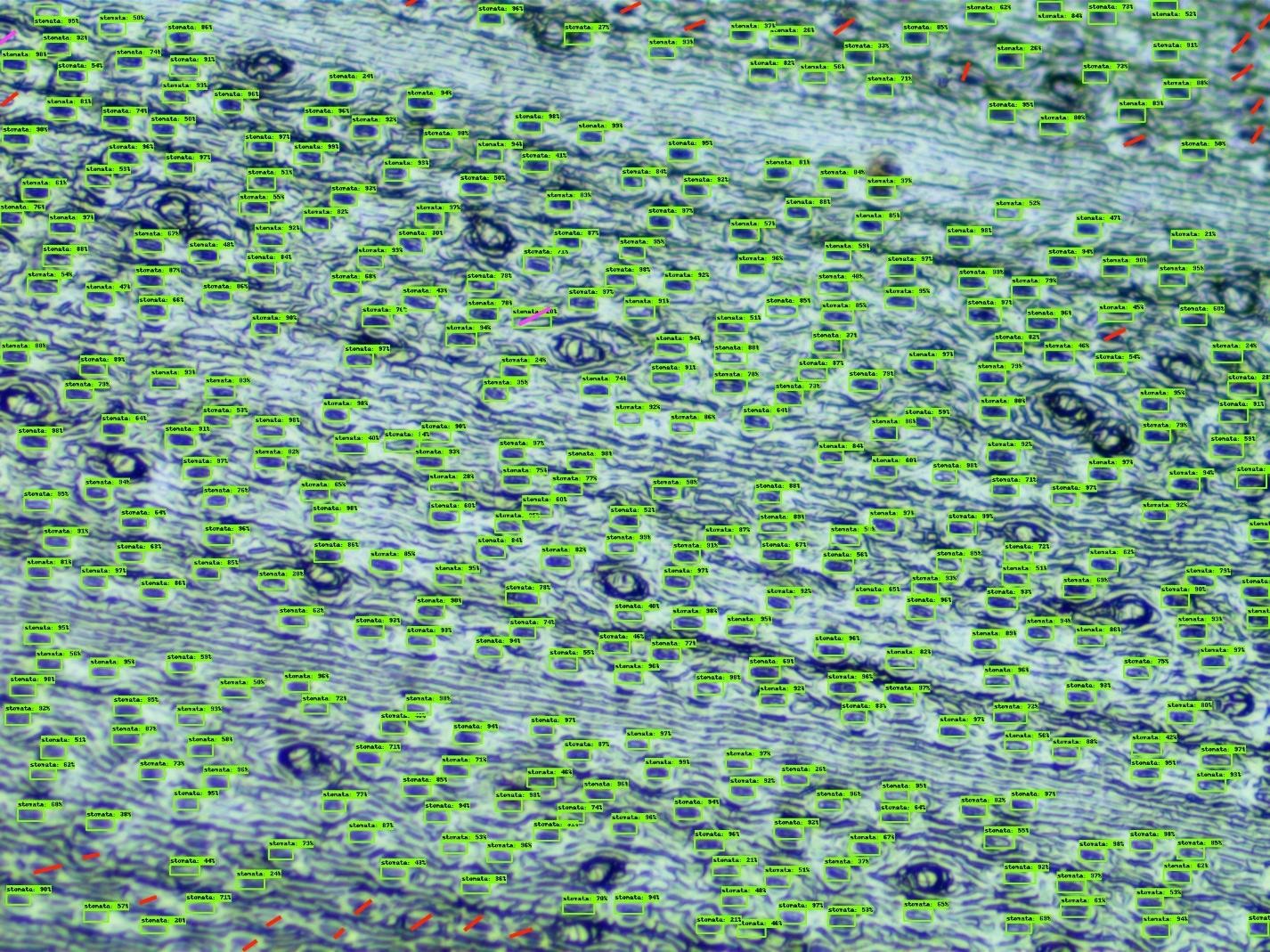


Supplementary Figure 4. Case with lowest accuracy for Set D. The stomata detection precision for this micrograph was 99.72%, and the recall was 93.71%. Both mislabeled and undetected stomata were manually marked in red. A single mislabeled stoma was found near the middle of the image. Most of undetected stomata were found at the unfocused top right or bottom left of the image.
